# Near-Surface Air Temperature Estimation Based on an Improved Conditional Generative Adversarial Network

**DOI:** 10.3390/s24185972

**Published:** 2024-09-14

**Authors:** Jiaqi Zheng, Xi Wu, Xiaojie Li, Jing Peng

**Affiliations:** Department of Computer Science, Chengdu University of Information Technology, Chengdu 610225, China; 3220608049@stu.cuit.edu.cn (J.Z.); wuxi@cuit.edu.cn (X.W.); lixj@cuit.edu.cn (X.L.)

**Keywords:** near-surface air temperature, conditional generative adversarial network, remote sensing, self-attention mechanism, multi-scale, deep learning

## Abstract

To address the issue of missing near-surface air temperature data caused by the uneven distribution of ground meteorological observation stations, we propose a method for near-surface air temperature estimation based on an improved conditional generative adversarial network (CGAN) framework. Leveraging the all-weather coverage advantage of Fengyun meteorological satellites, Fengyun-4A (FY-4A) satellite remote sensing data are utilized as conditional guiding information for the CGAN, helping to direct and constrain the near-surface air temperature estimation process. In the proposed network model of the method based on the conditional generative adversarial network structure, the generator combining a self-attention mechanism and cascaded residual blocks is designed with U-Net as the backbone, which extracts implicit feature information and suppresses the irrelevant information in the Fengyun satellite data. Furthermore, a discriminator with multi-level and multi-scale spatial feature fusion is constructed to enhance the network’s perception of details and the global structure, enabling accurate air temperature estimation. The experimental results demonstrate that, compared with Attention U-Net, Pix2pix, and other deep learning models, the method presents significant improvements of 68.75% and 10.53%, respectively in the root mean square error (RMSE) and Pearson’s correlation coefficient (CC). These results indicate the superior performance of the proposed model for near-surface air temperature estimation.

## 1. Introduction

Temperature is one of the most important parameters in meteorological data collection [1,2,3]. The near-surface air temperature, which is typically measured at a height of 1.5 to 2 m above the ground, reflects the ambient temperature [4]. As an indicator of surface radiation exchange and heat balance, it governs most land-surface processes [2,5]. This parameter, which signifies the warmth or coolness of the near-surface air, is closely associated with the growth and development of plants and animals, as well as human activities. Consequently, it serves as a crucial element in climate change research [6]. At present, near-surface air temperature data are primarily collected by meteorological observation stations, which measure atmospheric temperature at a height of 1.5 to 2 m near individual monitoring sites. However, the distribution of these observation stations is uneven, due to geographical and environmental factors, and they are particularly sparse or even absent in remote and geographically unique areas. This uneven distribution restricts the near-surface air temperature monitoring range, impeding the acquisition of high-resolution spatial temperature distributions and complicating the study of the spatial distribution and the internal structure of temperature levels in specific locations. These limitations hinder subsequent data analysis and applications [7,8,9,10].

In recent years, satellite remote sensing technology and associated operational products have significantly improved. Satellite remote sensing provides continuous observations with broad spatial coverage, minimal ground interference, and high spatial resolution, making it an effective method for addressing the limitations of observation stations. This technology offers a feasible way to obtain continuous spatiotemporal distribution information of near-surface air temperature [11,12]. As a result, the estimation of near-surface air temperature using remote sensing satellites has emerged as a novel approach, with methods classified into the following types, each possessing its own strengths and limitations.

Statistical methods utilize linear regression models to link remote sensing data with ground-based measurements [13], but their effectiveness is limited by the variability in meteorological station density and the high demand for input data, resulting in poor model portability [14]. The Temperature–Vegetation index (TVX) method estimates temperature by correlating normalized vegetation indices with surface temperature [15]; however, it struggles with accuracy in areas with sparse or no vegetation cover and relies heavily on empirical determinations [16]. The surface energy balance method models temperature through iterative calculations based on energy exchange principles [17], but its complexity and the requirement for difficult-to-obtain ground parameters often lead to error propagation and limited practical application [17]. Lastly, the atmospheric profile extrapolation method assumes continuous vertical temperature changes to interpolate near-surface temperatures [1], but it lags in accuracy compared to other methods, particularly under cloudy conditions where thermal infrared data cannot be effectively utilized [18,19].

In recent years, deep learning methods have brought about revolutionary changes in the field of meteorology, driven by their rapid development and powerful data processing capabilities [20]. With the advancement of satellite remote sensing technology and the onset of the big data era, numerous studies have started to use deep learning techniques to improve the prediction and estimation of meteorological elements. The strengths of these techniques, in terms of feature extraction and pattern recognition, have been leveraged to enhance the efficiency and accuracy of weather forecasting. These methods not only allow for the management of large-scale remote sensing data but also uncover deep patterns within the data, providing new perspectives and tools for meteorological research [21].

In 2016, Tao et al. [22] proposed the use of a stacked denoising autoencoder network to process homogeneous PERSIANN-CCS data for the error correction of PERSIANN-CCS precipitation products. The results demonstrated significant improvements in precipitation identification and rainfall intensity assessment with the application of this method. In 2017, Tao et al. [23] developed a deep learning network with two hidden layers that utilized 10.8 μm infrared and 6.7 μm water vapor channel images from the Geostationary Operational Environmental Satellite (GOES) as inputs to generate precipitation identification maps of the region as outputs. The experimental results indicated that the identification of precipitation through deep-learning-based feature extraction from cloud images significantly outperformed traditional manual feature extraction methods. In 2019, Sadeghi et al. [24] introduced a ten-layer convolutional neural network (CNN) for training and used water vapor and infrared channel images from the GOES as input data. They found that the CNN model surpassed PERSIANN-CCS and PERSIANN-SDAE in terms of precipitation identification accuracy and the correlation coefficient for precipitation prediction. In 2020, Shen et al. [25] proposed a deep belief network (DBN) that integrates remote sensing, socio-economic, and station data to estimate near-surface air temperature. In the same year, Jiang et al. [26] proposed a deep-learning-based method for reconstructing satellite data and supplementing missing radar echo data. In 2021, Wang et al. [27] combined data-driven and knowledge-based technology with deep learning models to develop a new method for overcoming the ill-posed problem of surface temperature retrieval. In 2022, Guo et al. [28] proposed a deep-learning-based rainfall classification method using synthetic aperture radar images, aiming to improve sea surface wind speed retrieval through integrating existing rainfall correction models.

Relevant research has clearly demonstrated the immense potential and significant advantages of deep learning in meteorology. However, several challenges and limitations persist in practical applications that cannot be overlooked. One area that needs more exploration is the estimation of the near-surface air temperature. Existing methods require improvements in accuracy and reliability, particularly when considering complex terrain and variable climate conditions. As a result, there is a need for the development of new deep learning architectures to improve the accuracy and generalization ability of near-surface air temperature estimation approaches in meteorological research.

In 2014, generative adversarial networks (GANs) were introduced [29]. GANs consist of a generator and a discriminator. The generator generates images from random noise, while the discriminator determines whether the input data are real or fake. The generator’s goal is to produce images that closely resemble real ones, attempting to fool the discriminator. In contrast, the discriminator aims to accurately distinguish between real and generated images. This adversarial process continues until an equilibrium is reached, where the discriminator can no longer distinguish between real and generated images. GANs offer several advantages, including the ability to train without labeled data, update gradients through backpropagation, and rapidly generate images [30]. These features have led to their widespread application in various fields, such as image generation, segmentation [31], inpainting [32], super-resolution [33], and denoising [34]. However, GANs also have limitations. The outputs are uncontrollable and lack interpretability due to the generation process being driven by random noise, requiring high consistency in the training data. Additionally, the original GAN is difficult to train and often encounters gradient vanishing issues when there is little overlap between generated and real samples. To address these challenges, numerous GANs variants have been developed.

Mirza et al. introduced the conditional generative adversarial networks (cGANs) in 2014 [35], which incorporated a conditional variable into both the generator and discriminator of the original GAN. By adding additional information to constrain the model, cGANs established a paired relationship between input and output, guiding the image generation process and resolving the issue of uncontrollable outputs. Unlike the original GAN, where the generator’s input was simply real or fake images, cGANs used a combination of real images with conditions or fake images with conditions. During the prediction, corresponding conditional information is inputted to obtain the desired output. Mirza and colleagues successfully applied cGANs to generate corresponding MNIST handwritten digit images by inputting random noise and one-hot encoded digits into the generator. The introduction of cGANs paved the way for the application of GANs in multimodal learning, leading to the development of many improved cGAN models.

In this study, a new method for near-surface air temperature estimation based on deep learning is proposed, specifically using an attention- and residual-enhanced multi-scale conditional generative adversarial network (ARM-cGAN). This method improves upon the conditional generative adversarial network (cGAN) framework through the creation of a generator that combines a U-Net backbone with a self-attention mechanism and cascaded residual blocks. The goal of this design is to selectively extract features that are crucial for temperature data generation while reducing redundant information. Additionally, a discriminator is designed to combine multi-scale spatial features at different levels, further improving the network’s ability to perceive details and overall structure, resulting in precise temperature estimation. The ARM-cGAN method effectively takes advantage of cGANs by incorporating conditional information to control the image generation process. It uses cloud image data from the FY-4A satellite as conditional information, along with data from channels 12 and 13, which effectively represent near-surface air temperature, including cloud and surface temperature. This guided approach helps to control the generation of temperature images, producing high-resolution spatio-temporal near-surface air temperature data and effectively addressing the problem of missing temperature data due to a lack of meteorological observation stations. The proposed ARM-cGAN method is compared with several deep learning models that have been proven to be effective in meteorological estimation and prediction tasks. The experimental results indicate that this method offers superior temperature estimation performance.

## 2. Study Area and Data

### 2.1. Study Area

This study focused on the geographical area ranging from 50° S to 50° N and 40° E to 140° E. This region was chosen for several reasons. First, it covers a wide range of climates, including sub-tropical, tropical, and temperate zones, as well as diverse geological features, such as mountains, plains, and lakes. Second, the area experiences significant seasonal variations, including warm summers and cold winters, which greatly influence the near-surface air temperature distribution. These variations make the temperature changes in this region representative. Additionally, this area is closely linked to the countries involved in the “Belt and Road” initiative. The research findings are expected to provide valuable scientific support for these countries in the context of meteorological forecasting, environmental monitoring, and climate change adaptation, giving practical significance to this study.

### 2.2. Fengyun-4A Satellite Data

The FY-4A satellite is a new-generation geostationary meteorological satellite developed in China. It is equipped with various observation instruments, including the Advanced Geostationary Radiation Imager (AGRI), the Interferometric Atmospheric Vertical Sounder, the Lightning Imager, and space environment monitoring instruments. The AGRI is one of the main payloads of FY-4A, which can perform precise and flexible two-dimensional pointing using a sophisticated dual-scan mirror mechanism, allowing for rapid regional scans at minute-level intervals. The AGRI uses an off-axis three-mirror optical system to capture Earth cloud images across 14 spectral bands and employs on-board blackbody calibration for frequent infrared calibration, ensuring the accuracy of the observational data [36].

The spectral range of the AGRI is from 0.45 to 13.8 μm, providing improved spectral resolution and measurement accuracy compared with the Visible and Infrared Spin Scan Radiometer (VISSR) on the FY-2 series. AGRI has 14 detection bands, including 2 visible bands, 1 near-infrared band, 3 shortwave infrared bands, 2 mid-wave infrared bands, 2 water vapor bands, and 4 longwave infrared bands. The detailed spectral band information and the corresponding detection targets are listed in Table 1. Channels 12 and 13 are known as atmospheric window channels, falling within the infrared radiation region and allowing the satellite to observe infrared radiation from the Earth’s surface. They are highly sensitive to temperature variations in the surface and cloud layers [37], providing high-quality information related to surface temperature. This study selected data from channels 12 and 13 of the AGRI sensor on the FY-4A satellite as input data, with a spatial resolution of 4 km.

### 2.3. ERA5 Reanalysis Data

The target temperature data used in this study were the ERA5 reanalysis data provided by the European Centre for Medium-Range Weather Forecasts (ECMWF). ERA5 is well known in the industry [38,39,40] and represents the latest generation of reanalysis data sets from the ECMWF. It offers significant improvements over its predecessor, ERA-Interim, including assimilation systems, model inputs, spatial resolution, output frequency, and data quality [41]. The ERA5 data set provides a spatial resolution of 0.25° × 0.25° and a temporal resolution of 1 h.

### 2.4. Data Preprocessing

The data used in this study came from channels 12 and 13 of the AGRI sensor on the FY-4A satellite, with ERA5 temperature data serving as the target temperature reference. The study area covered the geographical coordinates between 50° S and 50° N and between 40° E and 140° E. To make the spatial resolution of both datasets uniform (at 0.25° × 0.25°), we conducted bilinear interpolation, taking into account the fact that the initial resolution of FY-4A satellite cloud data was 4 km and that the initial resolution of ERA5 temperature data was 0.25° × 0.25°. To match the timing, we selected the FY-4A satellite data closest to the full hour, in order to align with the 1 h temporal resolution of the ERA5 data.

As shown in Table 2, the data set covered the period from 1 January 2020 to 31 December 2022, spanning a total of 36 months. A total of 25,380 data were utilized to create the training and testing sets. Specifically, 16,979 data from 2020 and 2021 were assigned to the training set. Of the remaining data from 2022 (a total of 8401 data across four seasons), 850 data were chosen randomly from each quarter, resulting in a total of 3400 data for the testing set. The ratio of the training set to the testing set was approximately 5:1, ensuring independence between the training and testing data to maintain the objectivity of the experimental evaluation.

## 3. Methodology

Generative adversarial networks (GANs), as introduced by Goodfellow et al. in 2014, are a type of generative model that learns through unsupervised training [29]. The GAN architecture is inspired by the concept of Nash equilibrium. The network learns the mapping from a random noise vector *z* to an output image *y* through the adversarial training of two modules: the generator and the discriminator, denoted as G:z⟶y. Expanding on this concept, Mehdi Mirza et al. proposed conditional generative adversarial networks (cGANs) in 2014 [35]. cGANs adopt a supervised learning approach, enabling the incorporation of additional conditional information to guide the generation process. By introducing conditional information into both the generator and discriminator, the outputs become more controllable and predictable. cGANs learn the mapping from the conditional information *x* and the random noise vector *z* to *y*, represented as $G:\left\{x,z\right\}⟶y$. The objective function of a cGAN is expressed as

(1) $$L_{cGAN}\left(G,D\right)=E_{x,y}\left[logD\left(x,y\right)\right]+E_{x,z}\left[log\left(1-D\left(x,G\left(x,z\right)\right)\right)\right]$$

where the symbol E represents the expectation operator, which is used in two different contexts. The first term denotes the expectation under the true data distribution for all pairs of real data and their corresponding condition (x,y), while the second term denotes the expectation under the noise distribution, which serves as input to the generator, for all pairs of generated data and their corresponding condition (x,G(x,z)). The generator *G* aims to minimize the loss L, while the discriminator *D* seeks to maximize L, leading to a competitive relationship between the two, which can be denoted as $arg\min_{G}\max_{D}L_{cGAN}\left(G,D\right)$.

Phillip Isola et al. [42] have demonstrated that, by including an L1 regularization loss in the generator’s loss function within the cGAN framework, the network can capture low-frequency information features and produce less blurry results. This significantly improves the mapping performance. Therefore, in this study, we use the loss function detailed in Equation (2):

(2) $$G^{*}=arg\min_{G}\max_{D}L_{cGAN}\left(G,D\right)+\lambda L_{L1}\left(G\right)$$

where the L1 loss is defined as

(3) $$L_{L1}\left(G\right)=E_{x,y,z}[||y-{G\left(x,z\right)||}_{1}]$$

In Equation (2), the first term represents the adversarial loss between the generator *G* and the discriminator *D*, which is in line with the cGAN objective function. The second term—the L1 pixel loss—evaluates the quality of the generator’s output. By optimizing the combined objective of adversarial loss and pixel loss, it is ensured that the generated images both have high realism and accurately reflect the mapping relationship with the input images.

**Figure 1:**
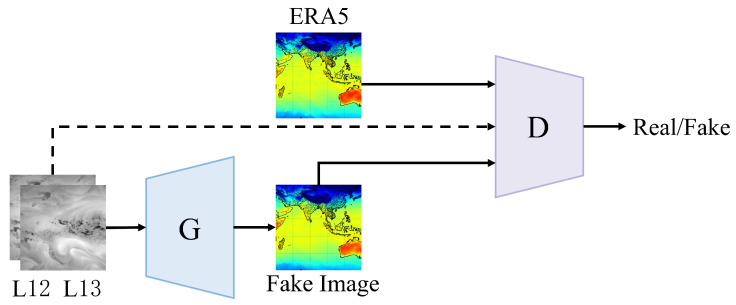
Structure of ARM-cGAN network.

The ARM-cGAN model’s overall structure is illustrated in Figure 1. The model starts by taking the dual-channel cloud image data (L12 and L13) from the FY-4A satellite and inputting them into the generator network *G* as conditional information. The generator then generates a synthetic “fake” temperature sample image. This generated image, along with the ERA5 temperature image, is combined with the original conditional information L12 and L13. These combined data are then fed into the discriminator network *D*, which evaluates whether the generated near-surface temperature image matches the real samples. Throughout this process, the generator and discriminator are trained adversarially, with the generator continuously improving to create more realistic temperature images, while the discriminator enhances its ability to distinguish between generated images and real samples. Ultimately, this adversarial training enables the generator to produce high-quality near-surface air temperature estimation samples.

### 3.1. Generator

**Figure 2:**
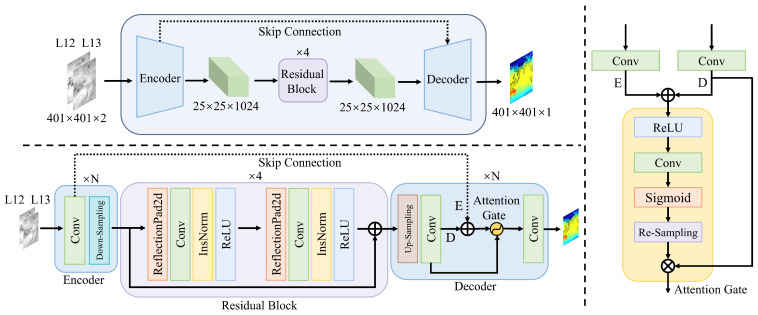
Structure of the generator network.

The generator structure of the ARM-cGAN network follows the U-Net’s encoder–decoder architecture, as depicted in the upper part of Figure 2. The encoder progressively reduces the input image size through a series of convolutional and pooling operations, thereby extracting essential features and decreasing the spatial dimensions. Throughout the encoding process, the output feature map at each level is transmitted directly to the corresponding level of the decoder via a skip connection, preserving the continuity and integrity of spatial information. On the other hand, the decoder gradually restores the image’s spatial resolution through up-sampling and convolution while receiving feature maps from the corresponding levels of the encoder through the skip connection. These feature maps, along with the up-sampling results of the decoder, are simultaneously input into attention gates to further refine and capture effective features, thereby enhancing the model’s reconstruction capabilities.

The ARM-cGAN model introduces four multi-scale residual modules between the encoder and decoder of the generator [43]. These modules deepen the network without changing the size and channel number of the input image. This expands the receptive field and improves the extraction of the correspondence between FY-4A dual-channel data and ERA5 temperature values. Each residual block consists of two 3 × 3 convolutional layers and employs a residual connection design, as depicted in the lower-left part of Figure 2. The feature maps output by the encoder serve as input to the residual blocks. Each convolutional layer applies edge reflection padding with a padding size of 1 pixel to maintain the feature map size and reduce edge effects, facilitating subsequent convolution operations while preserving spatial resolution. Each convolutional layer uses a 3 × 3 convolutional kernel with a step size of 1, keeping the dimensions of the input and output feature maps constant. Instance normalization is applied to normalize each channel of each sample independently after convolution, effectively maintaining sample independence and preserving the original details of the image. It is particularly suitable for processing cross-domain data such as temperature estimation tasks in remote sensing imagery missions. The activation function (ReLU) introduces non-linearity by retaining features when the input is greater than zero and setting the feature value to zero when it is less than zero. This allows the model to capture complex features, contributing to network sparsity and improving computational efficiency. Dropout is added between the convolutional layers (with a dropout rate of 50%) to enhance the generalization of the model. Finally, the input is added to the output of the two convolutional layers using identity mapping. Overall, the four residual blocks deepen the network and enhance the model’s representational capacity. This enables the model to learn structural information more effectively and recover key indicative features related to temperature, such as gradients and local anomalies. The residual connections also retain the original input information through skip connections, mitigating the gradient vanishing problem, increasing the network’s adaptability, and making the model better suited to variations in different meteorological data.

The self-attention mechanism is a technique that dynamically adjusts the weights based on the relationships between elements in the input sequence. When combined with attention gates and the U-Net architecture, the model can suppress irrelevant parts and enhance task-related features during the learning process, ultimately achieving better performance [44]. As the near-surface air temperature is influenced by various geographic and meteorological factors, the self-attention mechanism helps the model to identify key factors when estimating the temperature distribution, particularly in regions where surface cover and terrain diversity have significant impacts on the temperature. Therefore, this study incorporates attention gates into the decoder, as shown on the right side of Figure 2. This module receives feature maps from the encoder’s down-sampling and the decoder’s up-sampling processes. It first applies a 1 × 1 × 1 convolution operation. The convolution kernel has a size of 1 and a stride of 1 and does not use padding, applying weights to the feature channels. This operation compresses the number of input feature map channels into an intermediate feature dimension, reducing computational complexity and emphasizing important features. Additionally, a batch normalization layer is applied after the convolution to balance the feature channels, resulting in the feature maps *E* and *D*. The feature responses are then fine-tuned using ReLU and sigmoid activation functions, respectively, thereby generating attention weights for the feature maps of the encoder and decoder. Additionally, another 1 × 1 × 1 convolution operation is used to extract contextual information and further enhance the weights, ensuring that the model can capture significant features related to the temperature distribution. This process is followed by re-sampling to ensure that the spatial resolution of the attention weights matches the feature maps. Finally, the attention weights are multiplied by the feature maps, resulting in weighted features. Through this process, the feature maps are fused with the original feature maps, enabling the model to capture long-range dependencies between different features and causing the network to focus more on spatial features related to temperature distribution.

### 3.2. Discriminator

Oscillation and instability often occur during the cGAN training process, leading to issues such as mode collapse and non-convergence. These problems primarily arise from the imbalance between the convergence rates of the generator and the discriminator. This imbalance is particularly notable when the generator is tasked with translating across multiple classes of remote sensing images, as its convergence is significantly slower than that of the discriminator. On the other hand, the discriminator often reaches an optimal state early on and consistently distinguishes real from fake images effectively. Furthermore, as this study incorporates both global and local information into the generator, the discriminator—which typically operates at a single local scale—also faces an imbalance. Therefore, it is necessary to provide the discriminator with multi-scale information.

**Figure 3:**
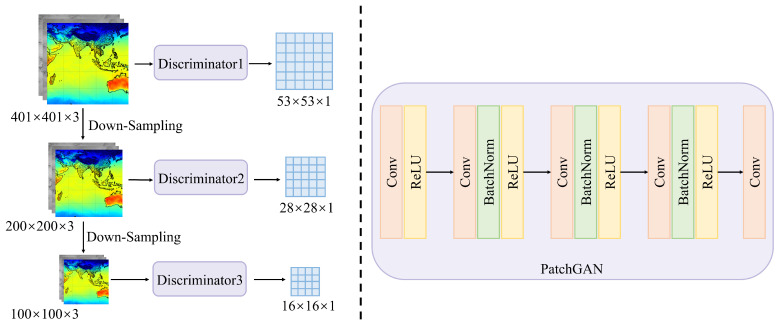
Structure of the discriminator network.

In this study, a multi-level and multi-scale discriminator network is introduced, as shown in Figure 3. Unlike the global-scale discriminators used in traditional GANs, this study proposes a pyramid-like multi-scale spatial feature fusion strategy for the discriminator. The network consists of three different levels of local-scale sub-discriminator networks cascaded within the discriminator. Initially, the input image pairs are down-sampled by factors of 2 and 4, and the resulting data are directed to corresponding sub-discriminators, namely, discriminator 1, discriminator 2, and discriminator 3. Each sub-discriminator shares a similar structure, as depicted on the right side of Figure 3. Through employing different scale factors, the sub-discriminator networks achieve receptive fields of 34 × 34, 68 × 68, and 136 × 136 on the input image pairs, allowing the discriminator to operate across various scales. After hierarchical features are extracted at different scales, the results from the three sub-discriminators are integrated to differentiate between real and generated input images. This design enhances the network’s perception of both detailed and global structures, guiding the generator to produce finer details. It also enables a comprehensive understanding of the input images, from the microscopic texture details of temperature variations to the macroscopic patterns of temperature distribution. Furthermore, the proposed discriminator network structure mitigates instability and mode collapse during network training, thereby improving the ARM-cGAN model’s ability to distinguish between generated and real images.

The structure of the sub-discriminator in this study uses a Markovian discriminator known as PatchGAN. PatchGAN is a fully convolutional network designed to penalize structures at the patch scale. In this study, the FY-4A dual-channel data are combined with the corresponding ERA5 temperature data and the generator output at the channel level, resulting in a 401 × 401 × 3 input data pair. After two down-sampling operations, the dimensions are reduced to 200 × 200 × 3 and 100 × 100 × 3, respectively. Subsequently, these three sets of data are processed by the corresponding sub-discriminators for evaluation. Each sub-discriminator uses five convolutional layers to extract high-frequency information from the images, ultimately mapping the input to an N × N matrix, in which each point represents the evaluation value for a small patch in the original image. The mean value of this matrix is then output as the final discrimination result. According to Phillip Isola et al. [42], N can be significantly smaller than the original image’s full size while still yielding high-quality results. Smaller PatchGANs have fewer parameters and operate more efficiently and can be applied to images of any size, addressing issues related to training instability and slow convergence. This characteristic makes them particularly well suited for handling remote sensing images with large amounts of high-resolution information.

## 4. Experiments and Results

### 4.1. Experimental Settings

The weights of the proposed improved conditional generative adversarial network (ARM-cGAN) were initialized following a normal distribution, and its parameters were optimized using the AdamW optimizer to minimize the model’s loss function. The loss function combined the cGAN loss with the L1 loss. The initial learning rate was set to 0.0002, and an adaptive algorithm (ReduceLROnPlateau) was employed to dynamically adjust the learning rate, reducing it by a ‘Factor’ of 0.5 after ‘Patience’ of 5 epochs without improvement. A batch size of 8 was used, and the model was trained for 50 epochs. Training was performed using a single NVIDIA GeForce RTX 3090 GPU utilizing PyTorch 1.8.0 and Python 3.8.

### 4.2. Comparison Models

U-Net: U-Net is a well-known neural network used in image processing. It was initially developed by Olaf Ronneberger et al. [45] for biomedical image segmentation. Its distinctive “U” shape consists of an encoder and decoder connected by skip connections, allowing it to effectively capture multi-level contextual information. U-Net has also been shown to be highly effective in tasks related to predicting and estimating meteorological elements, making it one of the most frequently used deep learning models in atmospheric science research [46].

TransUNet: TransUNet, an innovative model proposed by Chen et al. [47], incorporates the Transformer into the image segmentation architecture. The Transformer encodes CNN feature maps into context sequences, while the decoder upsamples these features and combines them with high-resolution maps for precise localization. TransUNet was applied by Yang et al. [48] to precipitation forecasting tasks. The study utilized precipitation data from the Royal Netherlands Meteorological Institute (KNMI) and compared the performance of TransUNet with other models, such as UNet, for precipitation forecasting. Through the incorporation of the Transformer encoder and attention mechanisms, TransUNet exhibited superior performance in capturing long-range dependencies and enhancing forecasting accuracy, especially with large-scale precipitation data.

Pix2pix: Pix2pix is an image-to-image translation model based on conditional GANs, which was introduced by Phillip Isola et al. [42]. It employs a straightforward and versatile processing architecture and loss function, enabling the generation of images or data with specific features based on given conditional information. Pix2pix has been extensively applied in areas such as image translation and super-resolution. Its robust style transfer capabilities make it particularly suitable for the temperature estimation task in this study.

Attention U-Net: The U-Net model has been improved through the addition of a self-attention mechanism, resulting in the creation of Attention U-Net. This enhancement, developed by Ozan Oktay et al. [44], enables the model to focus more effectively on important features, leading to improved prediction accuracy. Attention U-Net has been shown to outperform traditional U-Net in tasks such as medical image segmentation. In addition, Yanbo Gao et al. [49] have successfully applied Attention U-Net for precipitation estimation, demonstrating its effectiveness in identifying precipitation regions and accurately estimating rainfall.

PERSIANN-CNN: The PERSIANN-CNN, or precipitation estimation from remotely sensed information using artificial neural networks—namely convolutional neural network—is a deep learning model created by Sadeghi et al. [24] specifically for precipitation estimation. This model utilizes a convolutional neural network (CNN) to analyze satellite images, providing accurate precipitation estimates. Compared with other algorithms in the PERSIANN series and traditional multi-layer perceptron algorithms, PERSIANN-CNN is more effective in learning relationships between neighboring pixels, resulting in more precise precipitation estimates for each pixel.

DLPE-MS: The DLPE-MS (deep-learning-based multi-scale network for precipitation estimation) is an innovative deep learning algorithm developed by Guangyi Ma et al. [50]. This network utilizes a multi-scale approach to predict precipitation across the contiguous United States (CONUS) during the summer. The authors also introduced the balanced weight mean square error (BWMSE) loss function to address the underestimation of extreme rainfall, which is a common issue due to the infrequency of such events in the data. The model was shown to estimate precipitation in the study area within 0.19 s.

CRN: A cascaded refinement network (CRN) is a method for generating images based on the semantic layout of pixels, proposed by Qifeng Chen et al. [51]. This network consists of multiple convolutional refinement modules that are arranged sequentially, with each module progressively enhancing the image’s details based on the previous module’s output. This iterative refinement process allows the CRN to improve the quality and realism of the image while maintaining high resolution. A CRN is capable of generating realistic images from input semantic labels by learning the semantic layout and geometric structure information from these labels. During optimization, the network adjusts its parameters through back-propagation, learning feature regression against real images. The testing phase is not restricted by image style, making CRNs versatile in generating diverse image types.

### 4.3. Evaluation Metrics

In this study, we used evaluation metrics that are widely recognized in remote sensing image processing research [24,49], namely, the root-mean-square error (RMSE) and the Pearson correlation coefficient (CC). The RMSE measures the magnitude of error between actual and estimated data. In this study, it was used to assess the error between the model’s estimated temperature values and those of the ERA5 reanalysis data. The RMSE is expressed here in degrees Celsius (°C) and ranges from 0 to positive infinity. A smaller RMSE value indicates a smaller error, signifying better model performance. Conversely, a larger RMSE value denotes a larger error, indicating poorer model performance. The CC is a linear correlation coefficient that is used in this study to quantify the degree of correlation between the estimated temperature values and the ERA5 reanalysis data. The CC value ranges from −1 to 1, with negative values indicating a negative correlation and positive values indicating a positive correlation. The closer the absolute value of the CC is to 1, the stronger the correlation. The formulas for these metrics are as follows:

(4) $$CC=\frac{\sum_{i=1}^{n}\left({\hat{T}}_{i}-\bar{\hat{T}}\right)\left(T_{i}-\bar{T}\right)}{\sqrt{\sum_{i=1}^{n}({\hat{T}}_{i}-\bar{\hat{T}}{)}^{2}}\sqrt{\sum_{i=1}^{n}(T_{i}-\bar{T}{)}^{2}}}$$

(5) $$RMSE=\frac{1}{n}\sum_{i=1}^{n}\sqrt{\sum_{i=1}^{n}{\left(T_{i}-\bar{T}\right)}^{2}}$$

where $\bar{T}$ is the average of all target values $T_{i}$, and $\bar{\hat{T}}$ is the average of all predicted values $\hat{T_{i}}$. By comparing its RMSE and CC values with those of other advanced deep learning methods, the effectiveness of the proposed method in accurately estimating the near-surface air temperature can be validated [52,53].

### 4.4. Experimental Results and Analysis

In this research, we used the CRN model as a baseline to compare the performance of various temperature estimation models. The results, shown in Table 3, indicate that all models performed better than the CRN model in terms of the RMSE and CC. The ARM-cGAN model showed the best performance in both metrics, with an RMSE of 1.4815 °C, representing a 68.75% improvement over the CRN model and indicating a significant improvement in near-surface air temperature estimation accuracy. Additionally, the ARM-cGAN model achieved a CC of 0.9897, which was the highest among all models and demonstrated the strongest positive correlation with the reference temperature values, reflecting a 10.5316% increase. These results demonstrate the superior effectiveness of the ARM-cGAN model in near-surface air temperature estimation compared with the other models. Due to the substantial differences in the RMSE and CC values between the CRN and PERSIANN-CNN models and the other models, these two models were excluded from subsequent graphical comparisons.

**Figure 4:**
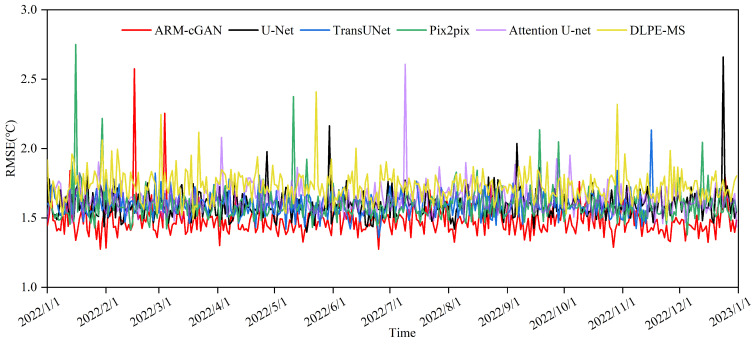
Time distribution of RMSE.

The graph in Figure 4 shows the daily variation in average RMSE across the test set for the ARM-cGAN, U-Net, TransUNet, Pix2pix, Attention U-Net, and DLPE-MS models. From January to June, the RMSE values fluctuated more frequently and widely, possibly due to the high atmospheric instability and significant temperature fluctuations, which are typical of spring. These conditions can lead to short-term temperature changes and extreme weather events such as cold fronts, warm fronts, and severe convection, making model predictions more complex. On the other hand, during the summer months (July to September), the RMSE values for all models stabilized and showed less fluctuation, which was likely due to the higher temperatures and increased atmospheric stability. Notably, the RMSE curve of the ARM-cGAN model consistently remained at the lowest level throughout the year, with smaller fluctuations in both range and frequency compared with the other models. This result highlights its effectiveness and robustness in temperature estimation.

**Figure 5:**
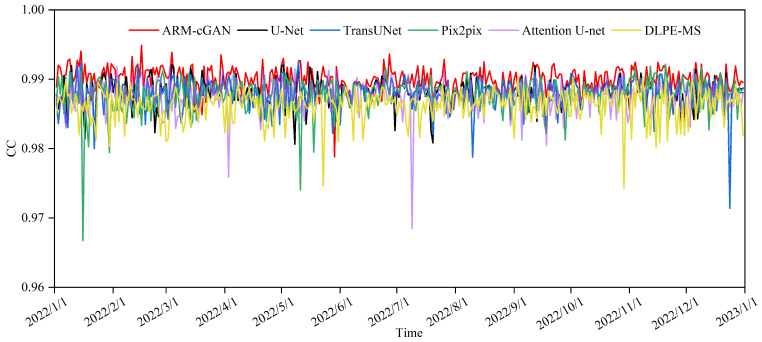
Time distribution of CC.

The graph in Figure 5 shows the daily variation in the average CC across the test set for the ARM-cGAN, U-Net, TransUNet, Pix2pix, Attention U-Net, and DLPE-MS models, where a higher curve indicates a stronger correlation between the model output and the ERA5 reference data. The graph indicates that, in June, all models exhibited a decrease in correlation, likely due to the climatic variability during this period. June is a transitional month marked by significant temperature fluctuations and an increase in extreme weather events, such as heavy rain and heat waves, which may lead to higher uncertainty in model predictions. Importantly, the CC curve of the ARM-cGAN model remained more stable overall and consistently closer to the ideal value of 1.00. Among the models, the ARM-cGAN model consistently ranked the highest, indicating that, despite the seasonal variations that impacted the performance of all models, the ARM-cGAN model maintained higher accuracy and robustness in temperature estimation across different seasons. This result demonstrated its superior consistency and stability compared with the other models.

**Figure 6:**
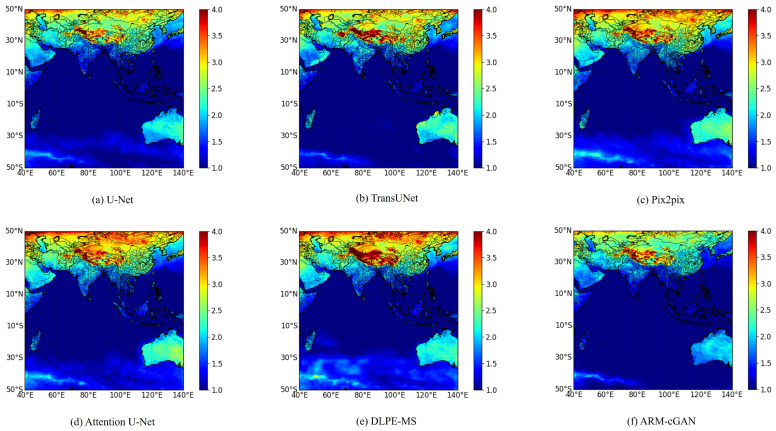
Spatial distribution of RMSE.

Figure 6a–f display the spatial distribution of the RMSE for each model on the test set. In the figures, warmer colors indicate higher RMSE values, suggesting larger estimation errors, while cooler colors signify lower RMSE values, indicating more accurate estimations. From Figure 6, it is evident that the DLPE-MS model exhibited warmer colors over land areas, indicating larger temperature estimation errors compared with the other four models. In contrast, the ARM-cGAN model generally showed cooler colors. In particular, in the regions of the Indonesian Archipelago (11° S–6° N, 94° E–141° E) and the island nation of Madagascar (12° S–25° S, 45° E–51° E), the ARM-cGAN model demonstrated significantly superior estimation performance. Additionally, in land regions such as Australia (10° S–42° S, 113° E–153° E) and the high-latitude areas around 50° N, the ARM-cGAN model achieved smaller errors and higher accuracy in estimating near-surface air temperature, confirming its robust performance across different geographical locations and climatic conditions.

**Figure 7:**
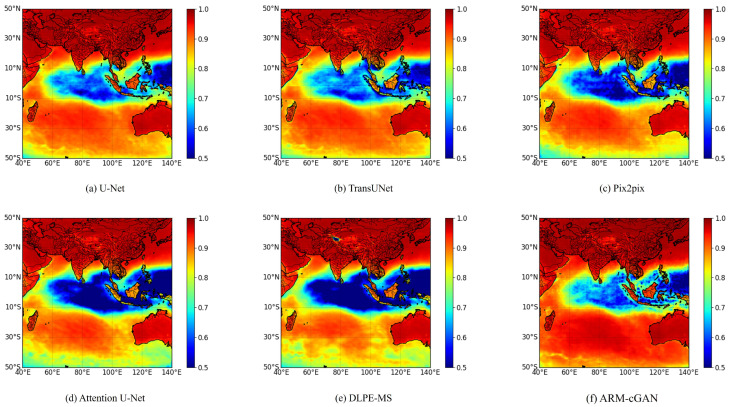
Spatial distribution of CC.

In Figure 7a–f, the spatial distribution of the CC for each model on the test set is shown. Warmer colors indicate a higher correlation between the model output and ERA5 reference data, while cooler colors represent weaker correlations. Figure 7 shows that all models displayed cooler colors in the region of the Indian Ocean near the equator (10° S–10° N) compared with land areas, indicating a decline in correlation within this area for all models. However, the ARM-cGAN model showed fewer cool colors in this region, compared with the other models, presenting a patchy color distribution, suggesting that it outperformed the other models in terms of temperature estimation accuracy even in challenging areas. Additionally, in low-latitude regions (30° S–50° S) and the Indonesian Archipelago (11° S–6° N, 94° E–141° E), the ARM-cGAN model exhibited noticeably warmer colors, indicating a higher correlation with the ERA5 reference temperatures. Overall, the ARM-cGAN model surpassed the other deep learning models in near-surface air temperature estimation across the entire study region. Its lower sensitivity to topographic and environmental changes indicates that ARM-cGAN can provide stable and reliable temperature estimates under diverse geographical and climatic conditions, highlighting its excellent generalization ability and further confirming the effectiveness and applicability of the ARM-cGAN model in meteorological estimation tasks.

**Figure 8:**
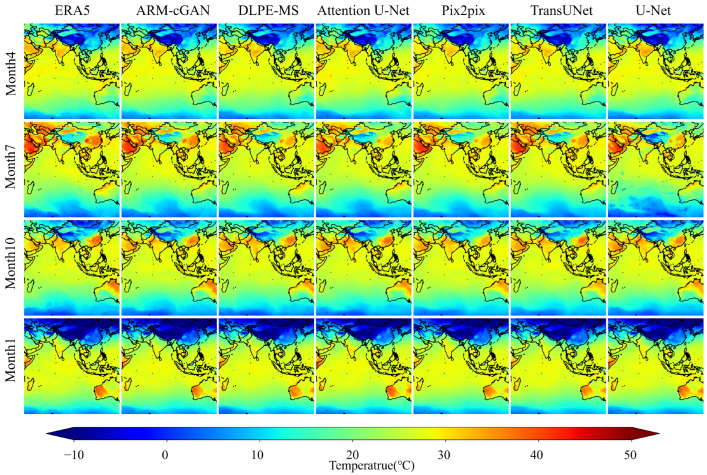
Effectiveness of different network models in estimating near-surface air temperature.

Figure 8 provides a visual comparison of temperature images for the same periods in January, April, July, and October on the test set, highlighting how well the different models estimated near-surface air temperature across seasons. The first column displays the true temperature images from ERA5 as the reference standard, while the following columns show the estimation results of the ARM-cGAN model and other models. Upon analyzing images from the four seasons, it is evident that the ARM-cGAN model consistently captured the temperature distribution, closely resembling the ERA5 data and outperforming the other models in terms of generalization and accuracy. The ARM-cGAN model accurately represented subtle seasonal climate effects and temperature fluctuations during critical transitions, particularly in July and October. For instance, in the temperature distribution map for July, the ARM-cGAN model closely estimated high temperatures in Southeast China, displaying a deeper red color that was similar to the true value from ERA5. In contrast, other models generally underestimated these temperatures. Additionally, for low temperatures observed in Southwest China, the ARM-cGAN model accurately presented a light blue color, reflecting the low temperatures in the region, while other models were less accurate. Even though the U-Net model performed well among the comparative models, it exhibited limitations in sensitivity to seasonal temperature fluctuations, with a deeper blue color in the temperature generation map for this period and region. These findings confirm the efficiency of the ARM-cGAN model in near-surface air temperature estimation across seasons, showcasing its reliability and applicability in handling complex meteorological data. This further validates its potential for use in meteorological estimation.

In conclusion, the ARM-cGAN model demonstrates robust and outstanding performance in near-surface air temperature estimation, as evidenced by the low RMSE values and high CC values presented in Figure 6 and Figure 7. This success is attributed to the cGAN framework employed by the ARM-cGAN model, which utilizes remote sensing satellite cloud images as conditional information to guide the generation of accurate temperature data under specific conditions. This approach not only overcomes the traditional GAN’s limitations—such as patternless image generation, low interpretability, and convergence challenges—but also effectively integrates cGAN with meteorological tasks, marking a significant achievement. The ARM-cGAN model combines a U-Net backbone with self-attention mechanisms and cascaded residual blocks in its generator, which are specifically designed to extract critical features for temperature data generation, thereby enhancing the network’s ability to reduce redundant information. Furthermore, a multi-scale spatial feature fusion discriminator enhances the model’s capacity to capture both fine details and global structures, resulting in precise temperature estimations. As shown in Figure 8, the model consistently provides accurate temperature estimations across various seasons and terrain types, underscoring its broad applicability in remote sensing data analysis.

### 4.5. Ablation Experiment

A series of ablation experiments were conducted on the ARM-cGAN model in order to verify the effectiveness of its core components. The experiments focused on three key components: cascaded residual blocks, the self-attention mechanism, and the multi-scale discriminator. Five variant models were constructed: a baseline model without any additional modules (Model 1), a model with cascaded residual blocks and the self-attention mechanism (Model 2), a model with cascaded residual blocks and the multi-scale discriminator (Model 3), a model with the self-attention mechanism and multi-scale discriminator (Model 4), and the full ARM-cGAN model incorporating all three components (Model 5). All the variant models were trained and evaluated on the same data set in order to ensure the fairness and comparability of the experimental results, which are presented in Table 4.

As shown in Table 4, compared with the baseline (Model 1), the introduction of any two components resulted in improvements in both key metrics: RMSE and CC. Notably, Model 2, which incorporated cascaded residual blocks and the self-attention mechanism, reduced the RMSE from 1.5956 °C to 1.5245 °C and improved the CC from 0.9880 to 0.9891. This indicates the significant contribution of these components to enhancing the temperature estimation accuracy. Model 3, which combined cascaded residual blocks and the multi-scale discriminator, maintained a low RMSE and slightly increased the correlation, suggesting that the discriminators enhanced the network’s ability to perceive both detailed and global structures. In Model 4, the combination of the self-attention mechanism and the multi-scale discriminator, though less impactful on the RMSE, still provided more refined discriminative capabilities, as reflected by the slight improvement in CC. The fully integrated Model 5—corresponding to the ARM-cGAN model proposed in this study—achieved the best performance among all variants. Additionally, an analysis of the experimental data revealed that all four models, each incorporating different module components, exhibited significantly improved accuracy in estimating extreme temperatures compared to the baseline Model 1, highlighting their performance advantage under extreme conditions.

In summary, the results of the ablation experiments clearly showed that each module component in the ARM-cGAN model was effective in contributing to its enhanced performance. Model 5, which combined all three key modules, not only demonstrated outstanding performance in regular temperature estimation but also exhibited exceptional accuracy and robustness under extreme temperature conditions, significantly improving the overall performance of the model. Specifically, the cascaded residual blocks improved the model’s learning capacity, the self-attention mechanism enhanced the recognition of key features, and the multi-scale discriminator increased the sensitivity to multi-scale feature recognition. The combination of these modules enabled the ARM-cGAN model to accurately estimate near-surface air temperature, validating the effectiveness of the proposed method.

## 5. Discussion

In this work, we propose an improved conditional generative adversarial network, called ARM-cGAN. It estimates near-surface air temperature in near real time using FY-4A satellite data, providing timely and accurate near-surface air temperature data for regions lacking station observations. A series of comparison and ablation experiments are conducted to verify the performance of ARM-cGAN using the operational product ERA5 as the benchmark standard. Based on the experimental results, our discussions are as follows.

Estimating near-surface air temperature from FY-4A satellite imagery based on deep learning is effective. This new method addresses the limitations of previous approaches that relied solely on remote sensing satellites for estimation. For example, statistical methods have poor model transferability due to variations in the density of ground meteorological stations. The TVX method is not suitable for regions with diverse terrain coverage. The surface energy balance method is complex and requires numerous parameters, while the atmospheric profile extrapolation method suffers from lower accuracy. In contrast, deep learning networks are known for approximating arbitrary functions with high accuracy. The proposed ARM-cGAN model uses the cGAN network architecture, incorporating cascaded residual blocks and a self-attention mechanism, and introduces a multi-scale discriminator to significantly enhance the accuracy of near-surface air temperature estimation using remote sensing satellite data. The model is structurally simple, has few parameters, and is not constrained by terrain, making it applicable to any region covered by remote sensing satellites. The effectiveness of the model was validated using two commonly employed evaluation metrics in temperature estimation tasks: RMSE and CC. In comparative experiments, the ARM-cGAN model outperformed other deep learning models effectively in meteorological element estimation and prediction tasks, achieving an RMSE of 1.4815 °C and a CC of 0.9897 compared to ERA5 reanalysis data. This demonstrates the model’s superior performance in near-surface air temperature estimation.

In addition, the selection of a suitable deep neural network can enhance performance. Unlike traditional deep learning networks, which are commonly used in meteorological tasks, this study exploits the unique benefits of the cGAN network for image generation. By effectively integrating these strengths with the specific requirements of temperature estimation, we achieve exceptional estimation performance. The proposed model expands the cGAN framework by creating a generator that combines a U-Net backbone with a self-attention mechanism and cascaded residual blocks. This design enables the model to selectively extract features that are essential for temperature data generation while enhancing the network’s ability to suppress redundant information. Furthermore, a multi-scale spatial feature fusion discriminator was developed to further enhance the network’s perception of both fine details and global structures, ultimately leading to precise temperature estimation. Ablation experiments conducted in this study validated the effectiveness of each module in the proposed task.

Moreover, it is important to consider that near-surface air temperature estimation can be influenced by meteorological conditions, seasonal fluctuations, and geographic variations. After thorough analysis, it has been found that the accuracy and consistency of temperature estimation, as measured by RMSE and CC values, can vary based on the month/season and location. For example, in August, RMSE values tend to be smaller and more stable, with higher and more consistent CC values during this period. Furthermore, regions at mid-to-high latitudes often show more diverse and unstable variations in RMSE and CC values. Considering these findings, future work should take into account factors such as latitude, longitude, topography, and climate to enhance the accuracy and reliability of near-surface air temperature estimation.

Lastly, although the ARM-cGAN model proposed in this study has demonstrated superior near-surface air temperature estimation capabilities, its generalization across different geographic regions and atmospheric conditions remains to be further explored. The model’s training and validation rely heavily on remote sensing data from the FY-4A satellite, and its observational range and resolution may limit global applicability. In regions where FY-4A data are unavailable or where its data quality differs significantly from other remote sensing sources, the model’s effectiveness may be compromised. This suggests that without access to FY-4A data, the model’s performance could degrade, restricting its broader applicability. Additionally, geographic variation and atmospheric conditions, such as complex terrain, cloudy weather, or extreme climates, may affect the quality of remote sensing data and, in turn, impact the accuracy of the model’s temperature estimates. While the ARM-cGAN model enhances adaptability to diverse meteorological conditions by incorporating a self-attention mechanism, cascaded residual blocks, and a multi-scale discriminator, its generalization performance still requires evaluation across more regions and climatic contexts. Future research should incorporate additional remote sensing data, such as MODIS or Sentinel satellite data, to assess the model’s performance under varying data conditions and explore its potential for global-scale meteorological forecasting and temperature estimation.

In conclusion, the ARM-cGAN model is currently limited to regions with FY-4A data. Future work should aim to expand its applicability and test its robustness and generalization across a broader range of geographic environments and atmospheric conditions.

## 6. Conclusions

Near-surface air temperature reflects the thermal characteristics of the air close to the ground and is a critical factor in the study of hydrology, ecology, climate dynamics, and processes such as vegetation photosynthesis, transpiration, and evaporation. Typically, near-surface temperature values are obtained from meteorological observation stations. However, geographic constraints make it difficult to acquire accurate temperature data with high spatial and temporal resolution.

This study addressed the challenge of near-surface air temperature estimation through the proposal of an improved conditional generative adversarial network, called ARM-cGAN. This model is based on a conditional GAN framework and incorporates several network modules, including cascaded residual blocks, a self-attention mechanism, and a multi-scale discriminator. Data from the FY-4A meteorological satellite were used as conditional guiding information, and ERA5 reanalysis data were used as target information for training. The experimental results demonstrated the model’s excellent performance and its advantages in terms of estimating near-surface air temperature, effectively improving the accuracy and quality of the estimated temperature data. This research not only provides an innovative tool for fields such as meteorology, ecology, and hydrology but also emphasizes the significant potential of deep learning in analyzing complex climate data.

Future work will concentrate on enhancing the model by including extra meteorological factors such as wind speed and direction. This will help to improve the accuracy of near-surface air temperature estimates, with the aim of developing a more comprehensive and precise temperature estimation model that can better meet the requirements of different fields relying on climate data analysis.

With the rapid development of remote sensing technology and deep learning, temperature estimation is poised for new opportunities and challenges. Future research can focus on the fusion of multi-source remote sensing data, such as integrating satellite data from MODIS, Sentinel, and Landsat, to improve the model’s generalization across diverse geographic regions and variable climate conditions globally. As the demand for real-time meteorological monitoring grows, model optimization and lightweighting will become key priorities. By employing model compression and optimization techniques, deep learning models can achieve efficient real-time temperature estimation, even in resource-limited environments. Additionally, combining global climate forecasting with regional micro-scale analysis to build a multi-scale climate prediction framework will enable a more precise capture of interactions between global climate changes and local characteristics. Future research should also aim to improve model interpretability, offering deeper insights into how the model extracts and estimates temperature features, thereby providing more reliable support for meteorological decision-making. In summary, the fusion of multi-source data, real-time model optimization, the integration of global and local analyses, and enhanced interpretability are critical future directions for advancing temperature estimation.

## Figures and Tables

**Table 1:** FY-4A satellite AGRI sensor main detection object and band information.

Channels Number	Band Range/μm	Center Wavelength/μm	Spatial Resolution/km	Primary Probe Object
1	0.45–0.49	0.47	1	aerosol
2	0.55–0.75	0.65	0.5	fog, clouds
3	0.75–0.90	0.825	1	vegetation
4	1.36–1.39	1.375	2	cirrus
5	1.58–1.64	1.61	2	snow
6	2.10–2.35	2.225	2	cirrus, aerosol
7	3.50–4.00	3.725	2	fire point
8	3.50–4.00	3.725	4	low clouds, fog
9	5.80–6.70	6.25	4	high-layer water vapor
10	6.90–7.30	7.1	4	mid-layer water vapor
11	8.00–9.00	8.5	4	low-layer water vapor
12	10.3–11.3	10.8	4	cloud and surface temperature
13	11.5–12.5	12.0	4	cloud and surface temperature
14	13.2–13.8	13.5	4	cloud-top height

**Table 2:** Distribution of training and test sets in FY-4A dataset.

Datasets	Years	Days	Quantities
Training	2020–2021	731	16,979
Test	2022	365	3400

**Table 3:** Summary of the performance of different deep learning models for near-surface air temperature estimation on the test set.

Methods	RMSE	CC	RMSE-Gain	CC-Gain
CRN	4.7355	0.8954	-	-
PERSIANN-CNN	4.3766	0.9136	7.5789%	2.0326%
DLPE-MS	1.7195	0.9861	63.6891%	10.1295%
Attention U-Net	1.6203	0.9876	65.7839%	10.2970%
Pix2pix	1.5956	0.9880	66.3055%	10.3417%
TransUNet	1.5768	0.9885	66.7026%	10.3976%
U-Net	1.5576	0.9887	67.1080%	10.4199%
ARM-cGAN	**1.4815**	**0.9897**	68.7450%	10.5316%

**Table 4:** Ablation experiment.

Model	Component			Evaluation Metrics	
	Cascaded Residual Blocks	Self-Attention Mechanism	Multi-Scale Discriminator	RMSE	CC
Model 1	-	-	-	1.5956	0.9980
Model 2	√	√	-	1.5245	0.9891
Model 3	√	-	√	1.5740	0.9883
Model 4	-	√	√	1.5806	0.9881
Model 5	√	√	√	**1.4815**	**0.9897**

## Data Availability

The FY-4A satellite data can be found at http://satellite.nsmc.org.cn/portalsite/default.aspx (accessed on 1 May 2023). The ERA5 data can be found at https://cds.climate.copernicus.eu/cdsapp#!/search?text=ERA5 (accessed on 5 July 2023).
